# Amplification of *Cyclin L1* is associated with lymph node metastases in head and neck squamous cell carcinoma (HNSCC)

**DOI:** 10.1038/sj.bjc.6602400

**Published:** 2005-02-08

**Authors:** C Sticht, C Hofele, C Flechtenmacher, F X Bosch, K Freier, P Lichter, S Joos

**Affiliations:** 1 Klinik für Mund-, Kiefer- und Gesichtschirurgie, Universitätsklinikum Heidelberg, Heidelberg, Germany; 2 Deutsches Krebsforschungszentrum Abteilung Molekulare Genetik (B060), Im Neuenheimer Feld 280, D-69120 Heidelberg, Germany; 3 Pathologisches Institut Universitätsklinikum Heidelberg, Heidelberg, Germany; 4 Molekularbiologisches Labor der Klinik für Hals-, Nasen-, Ohrenheilkunde, Universitätsklinikum Heidelberg, Heidelberg, Germany

**Keywords:** head and neck squamous cell carcinoma, oncogenes, *Cyclin L1*, tissue microarray analysis, FISH

## Abstract

Overrepresentation of chromosomal bands 3q25–q29 has been associated with shortened disease-specific survival in head and neck squamous cell carcinoma (HNSCC). To assess the prevalence of copy number gains (>4 signals per cell) and high-level amplifications (>8 signals per cell) from putative oncogenes in this chromosomal region (*CCNL1*, *SNO*, *PIK3CA*, *TP73L*), tissue microarray analysis was applied on 280 HNSCCs by fluorescence *in situ* hybridization. Overall frequency of additional copy numbers was 34.3% for *CCNL1*, 31.8% for *SNO*, 39.0% for *PIK3CA* and 38.3% for *TP73L*, respectively. In general, gains were more frequently detected in stage IV compared to stage I–III tumours. Performing multivariate logistic regression analysis, a significant association of *CCNL1* gains and the presence of lymph node metastases was found, which was independent of anatomical site and T-stage of the primary tumour (*P*=0.049). Site-specific subgroup analysis further showed that copy number gains of *CCNL1* and *SNO* occurred more frequently in oral carcinomas in advanced clinical stages as compared to N0 oral lesions (*CCNL1*: *P*=0.03; *SNO*: *P*=0.03). Finally, Kaplan–Meier analysis revealed that high-level amplifications of *CCNL1* correlated with shorter overall survival of the patients. Our results indicate that *CCNL1* plays a critical role in the loco-regional progression of HNSCC and may serve as an indicator for occult advanced tumour stages.

Squamous cell carcinoma of the head and neck (HNSCC) represents the sixth most common human neoplasm. Despite novel combined adjuvant and neoadjuvant therapeutic approaches, the 5-year survival rate did not exceed 55% during the last decade (Landis *et al*, 1999). Therapeutic decisions are usually based on clinicopathological parameters like TNM stage and histological grading, which, however, often fail to predict patients' outcome. Therefore, there is a need to better understand HNSCC development on the molecular level. This should lead to an improved stratification between higher- and lower-risk patients, which can be treated in a more selective and individualised manner. Furthermore, understanding molecular pathomechanisms might identify therapeutical interference points.

Copy number gain on chromosome 3 with a minimal overlapping area of bands 3q25-qter is a recurrent molecular alteration in HNSCC (Speicher *et al*, 1995; Weber *et al*, 1998). According to Bockmühl *et al* (2000), this aberration is correlated with a decreased overall survival. The corresponding chromosomal region harbors several potential proto-oncogenes, as for example, *TP73L*, *PIK3CA*, *SNO* and *CCNL1*, which may contribute to a more aggressive tumour progression. *TP73L* is a member of the *P53* gene family and found overexpressed in various HNSCC cell lines (Hibi *et al*, 2000). *PIK3CA*, which is involved in multiple cancer-related functions like cell survival, proliferation and cell migration (Iyer *et al*, 1999; Guhaniyogi and Brewer, 2001), was considered to play a role in early HNSCC tumorigenesis since genomic amplifications were detected in precancerous oral dysplasias (Redon *et al*, 2002). Increased expression of *PIK3CA*, however, failed to predict clinical outcome in a small collection of primary HNSCC (Yang *et al*, 1998). *SNO* (SKI-related novel gene) includes two distinct genes, *SNO*-A and *SNO*-N, the latter one being a component of the TGF*β*/SMAD pathway (Stroschein *et al*, 1999). Amplifications of the gene were previously detected in primary oesophagus squamous cell carcinoma (Imoto *et al*, 2001). Finally, *CCNL1* (also termed *Cyclin L1* and *Ania-6a*) codes for a putative key regulator of pre-mRNA processing and is involved in G0 to G1 transition during the cell cycle (Iyer *et al*, 1999; Berke *et al*, 2001). In a recent study, it has been shown to be located on a small region highly amplified in a HNSCC-derived cell line and to be overexpressed in HNSCC primary tumours (Redon *et al*, 2002).

In the present study, 280 clinically well-defined HNSCCs biopsies mounted on a tissue microarray were analysed for copy number changes of *CCNL1*, *SNO*, *PIK3CA* and *TP73L* by fluorescence *in situ* hybridisation (FISH). In this way, we wanted to assess the role of each of these candidate genes with regard to aggressive progression and unfavourable clinical outcome of HNSCC.

## MATERIAL AND METHODS

### Tumour material

In total, 280 primary, paraffin-embedded HNSCC, 124 oral squamous cell carcinomas (OSCC), 96 pharyngeal squamous cell carcinomas (PSCC) and 60 laryngeal squamous cell carcinomas (LSCC) were obtained from the archives of the Institute of Pathology, University of Heidelberg. For all tumours, histopathological and clinical follow-up data were available. Head and neck squamous cell carcinomas were graded according to the TNM system and the UICC stage. Uvula mucosa tissue from healthy donors was used as reference for FISH experiments. The study was approved by the Medical Ethics Commission, University of Heidelberg.

### TMA construction, FISH analysis and statistical evaluation

Generation of TMAs was performed as previously described (Freier *et al*, 2003). Briefly, HE-stained sections were cut from each block to define representative tumour regions. Small tissue cylinders with a diameter of 0.6 mm were taken from selected areas of each donor block using a tissue chip microarrayer (Beecher Instruments, Silver Spring, MD, USA) and transferred to a recipient paraffin block. In all, 280 different tumour specimens and 10 control samples were arrayed. The recipient paraffin block was cut in 5-*μ*m paraffin sections using standard techniques.

For FISH experiments, BAC clones RP11-555M1 (*CCNL1*), RP11-373I6 (*TP73L*), RP11-245C23 (*PIK3CA*) and RP11-543D10 (*SNO*), obtained from the Resource Center and Primary Database, Germany (RZPD), were used as probes. Clones were prepared from bacterial cultures using Qiagen-Plasmid-Kit® (Qiagen GmbH, Germany) and labelled by nick translation with cyanine-3-dUTP (Perkin-Elmer Life Science, Boston, MA, USA). As internal control, BAC clone RP11-101p149 (chromosome 12q24) was differentially labelled with fluorescein-12-dUTP and co-hybridised.

TMA slides were first deparaffinised in xylene, immersed in 0.2 N HCl, and incubated in 1 M sodium thiocyanate solution at 80°C for 30 min. Subsequently, they were digested in a protease solution (0.5 mg ml^−1^ in 0.9% NaCl, pH 2.0) for 20 min at 37°C. Slides were post-fixed in 10% buffered formalin for 10 min, dehydrated in ethanol and air-dried. For FISH experiments, 200 ng of labelled probe and control DNA, respectively, were added to a TMA slide in a hybridisation solution containing 50% deionised formamide, 10% dextran sulphate, 2 × SSC, 2 *μ*g salmon sperm DNA, and 10 *μ*g Cot-1 DNA. The TMA slide and probe DNA were denaturated at 75°C for 10 min and hybridised overnight in a humidified chamber at 37°C. Subsequently, the slides were washed three times in 50% deionised formamide/2 × SSC at 42°C for 10 min and three times in 2 × SSC at 42°C for 5 min. Interphase nuclei were counterstained with 0.5 *μ*g *μ*l^−1^ 4,6-diamidino-2-phenylindole (DAPI) in Vectashield mounting medium (Vector Laboratories, Burlingame, CA, USA).

For evaluation of the experiments, hybridisation signals from 25 nonoverlapping interphase cell nuclei of each tumour sample were counted using a fluorescence microscope. A copy number gain was scored, if the average number of signals per nucleus was between 4 and 8, while a high level amplification was defined as more than 8 signals per cell or if clusters of multiple signals were visible. Fisher's exact test and the *χ*^2^ test were performed in order to compare the prevalence of gene copy number gain according to anatomic site, T-stadium, stage and the presence of lymph node metastases. Multivariate logistic regression analysis using the statistical package SPSS (SPSS, Munich, Germany) was applied to all patients with complete clinicopathological data sets. For overall survival analysis, Kaplan–Meier curves of HNSCC subgroups were analysed by log rank test. To examine the relative impact of oncogene copy number gains on the overall survival of the patients, a Cox proportional hazards regression model was applied for each oncogene with anatomic site and UICC stage as co-variables. *P*-values ⩽0.05 were considered as statistically significant in all analyses.

## RESULTS

**Figure 1:**
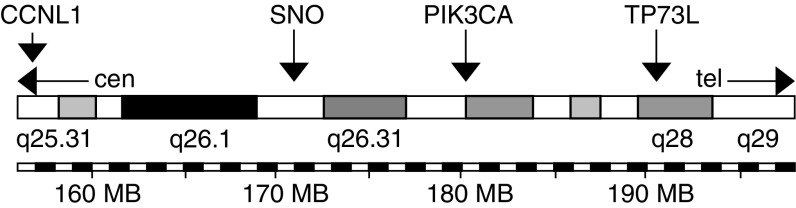
Localisation of the genes analysed on chromosome 3q25–q29.

**Figure 2:**
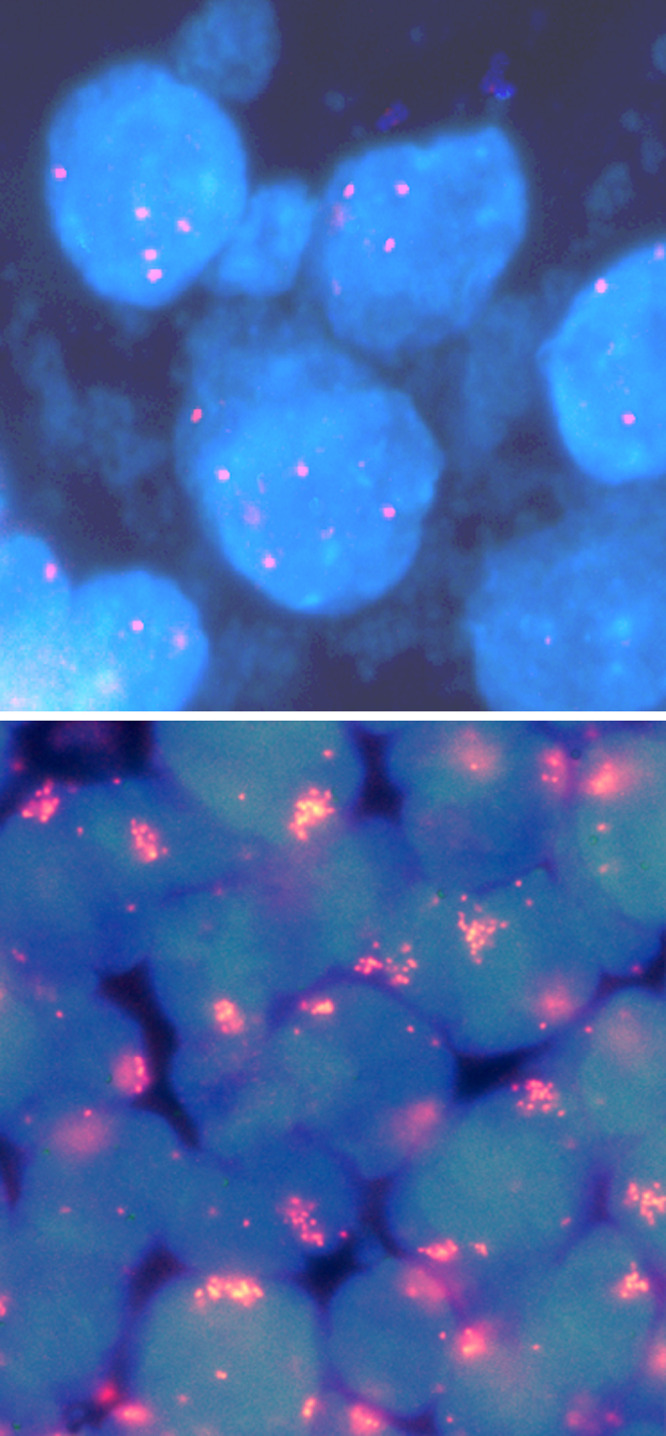
Detection of gene copy number gains by FISH in HNSCCs. Low-level copy number gain of *PIK3CA* (top) and high-level amplification of *CCNL1* (bottom).

A total of 280 HNSCC tumours from 280 patients were analysed by FISH for copy number changes of proto-oncogenes *CCNL1*, *SNO PIK3CA* and *TP73L* located on chromosomal band 3q25–q29 (Figures 1 and 2). The overall frequency and results from univariate statistical analyses are summarised in Table 1. A significant difference in the prevalence of numerical changes was observed in HNSCC of different anatomical sites, with PSCC exhibiting more copy number gains than LSCC and OSCC (*CCNL1* and *SNO*: *P*<0.001; *PIK3CA*: *P*=0.003; *TP73L*: *P*=0.007). With regard to the clinical stage, the prevalence was significantly higher in stage IV carcinomas as compared to stage I–III carcinomas (*CCNL1*: *P*<0.001; *SNO*: *P*=0.05; *PIK3CA*: *P*=0.009; *TP73L*: *P*=0.03). Finally, concerning lymph node metastases, *CCNL1* and *TP73L* showed an increased frequency of gains (*CCNL1*: *P*=0.02; *TP73L*: *P*=0.05), while for *SNO* and *PIK3CA* no such correlation was observed.

To create a multivariate model describing the risk for lymph node metastases, additional clinical parameters were investigated by univariate analysis. A significant association with the prevalence of lymph node metastases was found for higher T-stage (T3/4 *vs* T1/2; *P*⩽0.001), for younger age (<60 years *vs*>60 years; *P*=0.005) as well as for pharyngeal localisation *vs* oral cavity *vs* laryngeal localisation (*P*⩽0.001). These parameters as well as gain of *CCNL1* and *TP73L* were included in further multivariate analyses. The backward and the forward procedures for variable inclusion/exclusion within the logistic regression model determined pharyngeal localisation (*P*⩽0.001), higher T-stage (*P*⩽0.001) and *CCNL1* copy number gain (*P*=0.049) as independent predictors for metastasis formation in HNSCC.

**Figure 3:**
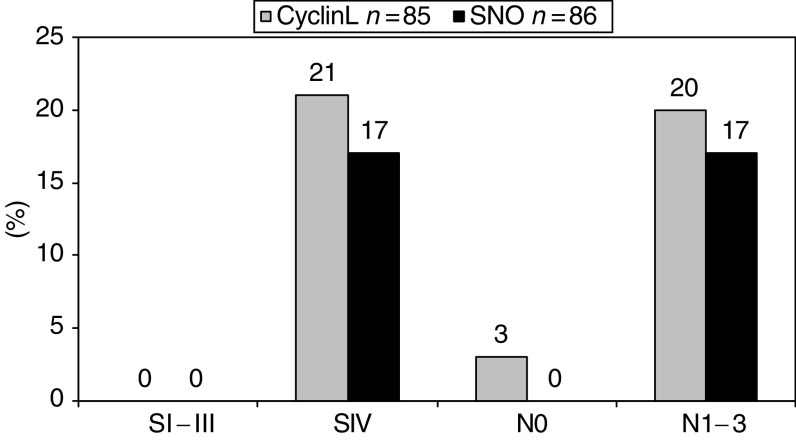
Frequency of *CCNL1* and *SNO* copy number gains in OSCC (SI–III and SIV: *CCNL1 P*=0.02, *SNO P*=0.03; N0 *vs* N1–3: *CCNL1 P*=0.05, *SNO P*=0.05).

Since HNSCC within the pharynx, lanrynx or oral cavity are different with regard to several clinical parameters (see below), a detailed analysis of the respective genes was performed in OSCC, PSCC and LSCC, separately. In OSCC (*n*=85), *CCNL1* and *SNO* copy number gains were primarily detected in tumours exhibiting lymph node metastases (*CCNL1*: *P*=0.03; *SNO*: *P*=0.03). In addition, gains of these genes occurred at higher frequencies in stage IV OSCC as compared to stage I–III OSCC (*CCNL1*: *P*=0.02; *SNO*: *P*=0.03) (Figure 3). No such association for any of the oncogenes was found in PSCC (*n*=69) and LSCC (*n*=41) (data not shown).

Regarding the clinical outcome, a significant correlation between shortened overall survival and higher T-stage (T3/4 *vs* T1/2; *P*⩽0.001), tumour site (pharyngeal *vs* laryngeal *vs* oral cavity; *P*=0.008) and advanced N-stage (N1–3 *vs* N0; *P*⩽0.001) was found in Kaplan–Meier analysis, whereas no such association for gains of any of the candidate genes was detected (*P*>0.05). These results were confirmed by multivariate survival analyses including the genes' numerical status, N-stage (N0 *vs* N1–3), T-stage (T1/2 *vs* T3/4), tumour site (pharyngeal *vs* laryngeal *vs* oral cavity) and age in the Cox proportional hazard models, where only the presence of lymph node metastases (*P*⩽0.001) and higher T-stage (*P*=0.009) were significant predictors of shortened overall survival.

**Figure 4:**
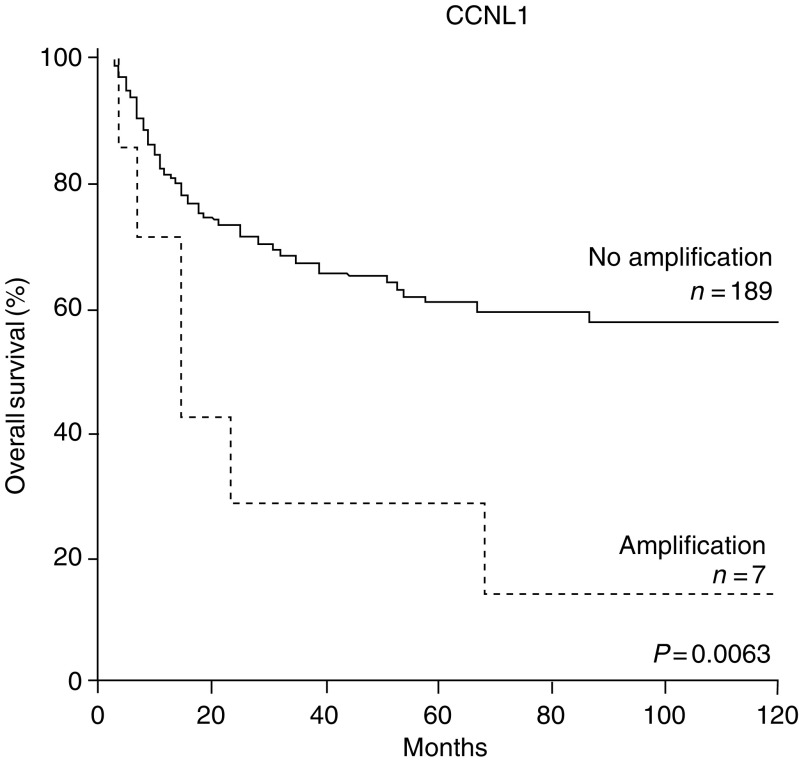
Kaplan–Meier survival analysis of HNSCC with or without *CCNL1* high-level amplification.

Since high-level amplifications might have a stronger impact on tumour formation and biological behaviour than low-level copy number gains (for definition, see Material and Methods), their influence on the clinical course was analysed separately. Although the absolute number of high-level amplifications of the genes analysed was rather low (see Table 1), a correlation with unfavourable clinical course in Kaplan–Meier analysis was found for *CCNL1* (log-rank test, *P*=0.006, Figure 4), but not for the remaining genes (all *P*-values >0.05).

## DISCUSSION

In the present study, the tissue array approach was applied in order to analyse copy number gains of putative oncogenes on a collection of 280 clinically well-documented HNSCC. We found significant associations between clinical parameters and copy number gains of four candidate genes, *TP73L*, *PIK3CA*, *SNO* and *CCNL1* located on chromosomal arm 3q. In general, frequencies of copy number gains were between 31.8% (*SNO*) and 39.0% (*TP73L*). Pharyngeal tumours (PSCC) showed the highest frequency in this respect, and this was significant on the univariate as well as on the multivariate levels (i.e. independent of the tumour size and metastasis status). It is well known that, in contrast to laryngeal and OSCCs, PSCCs are more aggressive, since they exhibit a higher tendency to metastasise and are associated with decreased overall survival (Baatenburg de Jong *et al*, 1993). A dosage effect of the analysed genes might therefore well contribute to this aggressive phenotype.

One of the major aims of this study was to search for a molecular marker, which is associated with a higher tendency of lymph node metastasis formation. Such a marker should be highly useful during the initial tumour staging and might give additional information concerning the presence of occult metastases. We found *CCNL1* to be more frequently increased in copy number in HNSSC with loco-regional metastases, irrespective of anatomic site and T-stage. Such an impact of *CCNL1* was also detected in OSCCs when analysed separately from PSCC and LSCC, suggesting that *CCNL1* could serve as a molecular indicator for loco-regional metastasis formation in HNSCC. In order to confirm this, however, further prospective studies have to be performed.

*CCNL1* was also interesting with respect to overall survival, in that high-level gene amplification (>8 signals/cell) was primarily detected in patients with lower survival rates. It should be noted that high-level amplification of *CCNL1* was found to be a rather rare event and therefore the number of cases is still low, not allowing a more rigid multivariate testing. Of note, this result shows that high-level amplifications of individual genes, in contrast to low-level gains, contribute to a distinct clinical behaviour and therefore both of these parameters might be considered separately in this type of analysis.

The molecular mechanisms which promote tumour progression by *CCNL1* remain to be determined. Up to now, only little is known about its function. *CCNL1*, like the closely related protein *CCNL2*, contain a N-terminal cyclin box but differs from the other members of the cyclin family by the presence of a so-called RS-domain, which is a hallmark of proteins involved in pre-mRNA processing (de Graaf *et al*, 2004; Yang *et al*, 2004). Both proteins were recently found to be colocalised with the splicing factor SC35 within nuclear speckles. *CCNL1* was also identified as an immediate early gene after induction of several growth factors including epidermal growth factor (Berke *et al*, 2001), and there is evidence that it is involved in G0 to G1 cell cycle progression (Iyer *et al*, 1999). Recently, *CCNL1* has been detected within a small amplicon on chromosome 3q25.3 in the HNSCC cell line CAL 27. Subsequent expression analyses revealed that it is recurrently overexpressed in primary HNSCC (Redon *et al*, 2002). Our data underline the pathogenic role of *CCNL1* in HNSCC progression, since an increased copy number of this gene was associated with the formation of loco-regional metastases and an unfavourable clinical outcome.

## Figures and Tables

**Table 1:** Frequency of copy number gains of *CCNL1*, *SNO*, *PIC3CA* and *TP73L* as well as corresponding *P*-values for univariate subgroup analysis

		***CCNL1***	***SNO***	***PIK3CA***	***TP73L***
HNSCC	*n*=280	34.3% (74/216); (3.2%) (7/216)	31.8% (69/217); (2.8%) (6/217)	39.0% (85/218); (6.0%) (13/218)	38.3% (86/224); (3.1%) (7/224)
OSCC	*n*=124	13.5% (13/96)	11.2% (11/98)	26.4% (24/91)	28.0% (28/100)
PSCC	*n*=96	56.2% (41/73)	49.3% (34/69)	51.3% (41/80)	51.4%) (38/74)
LSCC	*n*=60	42.6% (20/47)	48.0% (24/50)	42.6% (20/47)	40.0% (20/50)
*P*-values		<0.001	<0.001	0.003	0.007

T1/2	*n*=101	27.3% (21/77)	28.8% (23/80)	30.4% (24/79)	33.7% (29/86)
T3/4	*n*=148	39.8% (47/118)	33.6% (39/116)	43.9% (50/114)	41.0% (48/117)
*P*-values		0.091	0.533	0.071	0.309
					
N0	*n*=80	23.5% (16/68)	28.1% (18/64)	30.5% (18/59)	28.6% (20/70)
N1–3	*n*=169	40.9% (52/127)	33.3% (44/132)	41.8% (56/134)	42.9% (57/133)
*P*-values		0.018	0.515	0.151	0.049
					
SI-III	*n*=75	19.0% (12/63)	21.9% (14/64)	23.6% (13/55)	26.9% (18/67)
SIV	*n*=174	42.4% (56/132)	36.4% (48/132)	44.2% (61/138)	43.0% (59/136)
*P*-values		0.001	0.049	0.009	0.031

The numbers in parentheses in the first line represent the number of tumours with distinct high-level oncogene amplification in all HNSCC.
